# Evaluation of Insertion Energy as Novel Parameter for Dental Implant Stability

**DOI:** 10.3390/jcm9092977

**Published:** 2020-09-15

**Authors:** Tanja Grobecker-Karl, Anthony Dickinson, Siegfried Heckmann, Matthias Karl, Constanze Steiner

**Affiliations:** 1 Department of Prosthodontics, Saarland University, Kirrberger Strasse 100, 66421 Homburg, Germany; Tanja.grobecker-karl@uks.eu (T.G.-K.); Siegfried.heckmann@uks.eu (S.H.); Constanze.steiner@uks.eu (C.S.); 2 Private Prosthodontic Practice, 1564 Malvern Road, Glen Iris 3146, Australia; ajd1@i-pros.com.au

**Keywords:** insertion energy, insertion torque, primary implant stability, resonance frequency analysis, surgical protocol

## Abstract

Insertion energy has been advocated as a novel measure for primary implant stability, but the effect of implant length, diameter, or surgical protocol remains unclear. Twenty implants from one specific bone level implant system were placed in layered polyurethane foam measuring maximum insertion torque, torque–time curves, and primary stability using resonance frequency analysis (RFA). Insertion energy was calculated as area under torque–time curve applying the trapezoidal formula. Statistical analysis was based on analysis of variance, Tukey honest differences tests and Pearson’s product moment correlation tests (α = 0.05). Implant stability (*p* = 0.01) and insertion energy (*p* < 0.01) differed significantly among groups, while maximum insertion torque did not (*p* = 0.17). Short implants showed a significant decrease in implant stability (*p* = 0.01), while reducing implant diameter did not cause any significant effect. Applying the drilling protocol for dense bone resulted in significantly increased insertion energy (*p* = 0.02) but a significant decrease in implant stability (*p* = 0.04). Insertion energy was not found to be a more reliable parameter for evaluating primary implant stability when compared to maximum insertion torque and resonance frequency analysis.

## 1. Introduction

Recent finite element analysis [1] and in-vitro experiments [2] have shown that trabecular bone definitely contributes to the insertion process and resulting implant stability. Consequently, surgical technique should be customized based on the bone quality found in the specific site and with consideration of the impact of different drilling protocols, implant designs [3,4], and implant material [5].

Several in-vitro studies have demonstrated that under-preparation of the osteotomy increased primary stability at the time of implant installation [6,7,8], however an increase in bone density has been shown to have a greater effect on the primary stability of the implant [9]. In an animal trial, Marin et al. [10] demonstrated that under-sizing an implant osteotomy led to an increase in insertion torque and had no detrimental effect on osseointegration when compared to wider bone preparation, despite substantial differences in the healing mode. On the other hand, Cha et al. [11] found that an increase in insertion torque caused microfractures in bone and resulted in increased resorption in the site. This observation is supported by two animal studies demonstrating that the speed of bone formation was lower, whilst marginal bone loss was greater [12], and an associated loss in implant stability [13] in undersized as compared to enlarged osteotomies. In a clinical study comparing the primary stability of straight-walled and tapered implants using an insertion torque device, the authors found higher levels of primary stability for tapered implants, but a resultant higher failure rate for this implant design. They concluded that the high insertion torque caused the destruction of peri-implant bone and compromised osseointegration [14].

Several parameters have been used for determining the level of primary implant stability; these include insertion torque [2,6,8,14,15], implant stability quotient [4,8,15], and removal torque [8,13]. From a biomechanical perspective, it has been argued that a ‘dynamic’ parameter, i.e., ‘work’ or ‘energy’ may be better suited for characterizing bone-to-implant interfacial strength [16]. With modern surgical motors being capable of accurately measuring torque values, several authors have been reporting ‘insertion energy’—defined as the area under a torque–time curve, as a measure for primary stability [17,18,19,20,21,22].

The aim of this in-vitro study was to determine how implant-related parameters (length, diameter) and surgical protocols affect the parameters ‘insertion energy’ and primary implant stability for one specific bone-level implant design.

## 2. Materials and Methods

**Figure 1:**
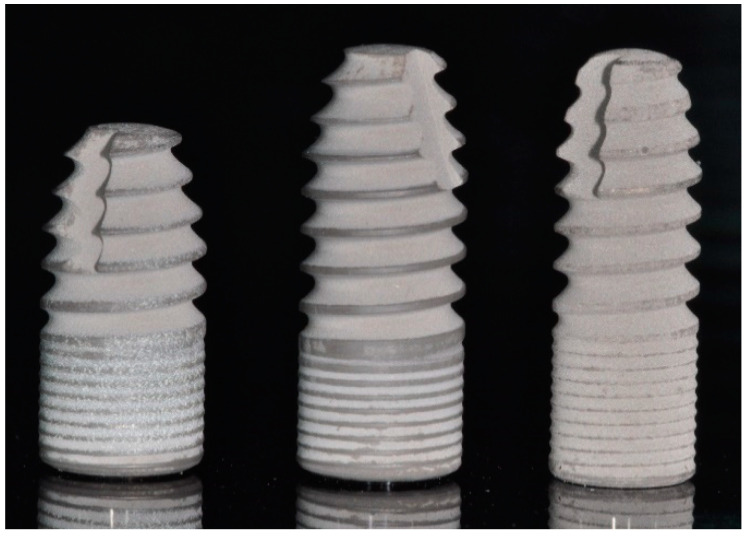
Implant dimensions used in this study: (**left**) implant dimension 4.8 × 10 mm; (**middle**) implant dimension 4.8 × 12.5 mm; (**right**) implant dimension 4.1 × 12.5 mm.

**Figure 2:**
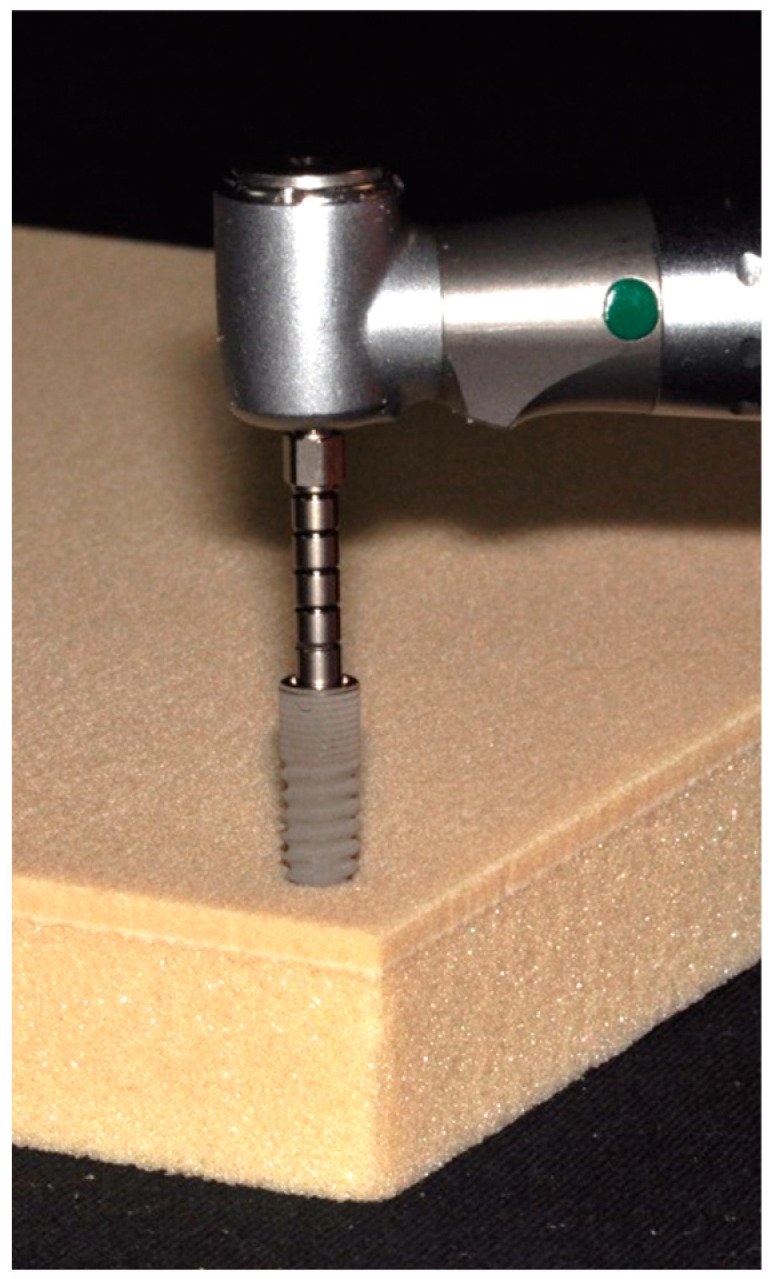
Implants were inserted in laminated polyurethane foam material using a surgical motor capable of recording the actual torque applied over time.

Twenty bone level implants belonging to one specific system (ICX, Medentis Medical GmbH, Ahrweiler, Germany) were divided into four groups (*n* = 5). The implants differed with respect to diameter and length (Figure 1; Table 1), with one group of implants (4.8 mm diameter and 12.5 mm length) acting as the control. The implants were placed in prepared sites in polyurethane foam blocks (Figure 2) consisting of a ‘trabecular’ portion covered by a ‘cortical’ layer with a thickness of 2 mm (Solid Rigid polyurethane foam 10 pcf/30 pcf, Sawbones Europe AB, Malmö, Sweden) [2,8,9,19]. The implant site preparation was completed using the manufacturer’s surgical drills and according to the manufacturer’s recommended drilling protocol for a medium bone density. In one group, the implants having the same dimensions as the control group, the drilling protocol was altered to that recommended for dense bone (Table 1). In the Control group, 4.8 × 12.5 mm implants were used, while in the Short group, implant length was reduced to 10 mm. In the Slim group, 4.1 × 12.5 mm implants were considered. For all of these groups, the osteotomies were prepared according to the medium bone drill protocol, while in the Dense group, 4.8 × 12.5 mm implants were placed following site preparation according to the dense bone protocol.

The implants were inserted into the prepared site with a surgical motor (speed set at 25 rpm) capable of recording actual torque over time (iChiropro, BienAir, Biel, Switzerland). Insertion torque was recorded with a sampling rate of 1/200 ms and exported into an Excel file, which could then be used for calculating the area under the curve applying the trapezoidal formula. Using implant specific MultiPeg abutments (Integration Diagnostics Sweden AB, Gothenburg, Sweden) and an Osstell ISQ device (Osstell AB, Gothenburg, Sweden), resonance frequency analysis (RFA) was used for quantifying primary implant stability in two directions perpendicular to each other [4,8,15].

Statistical analysis comparing maximum insertion torque, primary implant stability, and insertion energy was based on analysis of variance. For pairwise comparison of means, the Tukey honest differences test was applied. In addition, the dataset was checked for potential correlations among parameters using Pearson’s product moment correlation test. The level of significance was set at α = 0.05 for all statistical operations conducted. Due to a lack of data on the specific implant system and the bone surrogate material in this study, a sample size calculation could not be performed.

## 3. Results

**Figure 3:**
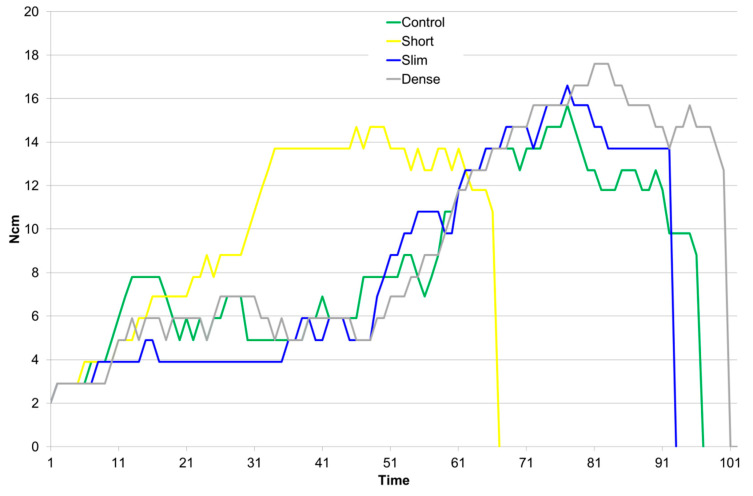
Overview of characteristic torque–time curves determined for the different implants and surgical protocols. Each curve represents one randomly chosen sample per group.

Despite an identical insertion speed of 25 rpm, differences were noted in the time needed for seating the different implant types, as seen in the torque–time curves recorded by the surgical motor (Figure 3). Implants of 10 mm length required less time to insert compared to 12.5 mm long implants, for both 4.1 mm and 4.8 mm diameters. The torque–time curves showed a reasonable level of reproducibility as did all other measurements and what led to standard deviations for all parameters below 15% (Table 2). The Shapiro-Wilk test confirmed the data obtained could be assumed to be normally distributed.

Analysis of variance (Table 3) revealed significant differences among groups with respect to implant stability (*p* = 0.01) and insertion energy (*p* < 0.01) but not for maximum insertion torque (*p* = 0.17). The use of 10 mm implants did not significantly alter maximum insertion torque (*p* = 0.82) but led to a significant decrease in implant stability (*p* = 0.01) compared to the 12.5 mm length implants. Reducing the implant diameter from 4.8 mm to 4.1 mm did not cause any significant change in the parameters tested, as compared to the control implants. The implants inserted using the drilling protocol for dense bone resulted in significantly higher values for insertion energy as compared to all the other groups (Control vs. Dense *p* = 0.02; Short vs. Dense *p* < 0.01; Slim vs. Dense *p* < 0.01). While not causing a statistically significant increase in maximum insertion torque (*p* = 0.49), the drilling protocol for dense bone led to a statistically significant decrease in implant stability (*p* = 0.04).

Comparing all data across the four different groups, good correlation was observed between maximum insertion torque and insertion energy (r = 0.75; *p* < 0.01). However, no correlation was observed between ISQ and insertion energy (r = 0.02; *p* = 0.95) nor between ISQ and maximum insertion torque (r = −0.10; *p* = 0.67). When considering the four experimental groups independently, no significant correlation across the parameters measured was observed (Table 4).

## 4. Discussion

A potential paradigm shift in surgical implant placement appears evident, transitioning from high insertion torque values being considered beneficial for mechanical primary implant stability [7,23], to an altered osteotomy and implant installation protocol to provide for a biologically acceptable level of primary stability—a successful outcome (osseointegration) that avoids damage to the jaw bone due to mechanical stress [10,11,12,13,24,25]. In evaluating this shift, insertion energy of a dental implant has been advocated as a relevant measure [17,18,19,20,21,22].

Based on the limited correlations of insertion energy with other parameters found in this study, it appears that this novel measure lacks advantages as compared to traditional measurements of implant stability. This assertion is further supported by the fact that only the dense bone protocol group demonstrated a significant difference in outcome with respect to insertion energy. A change in the implant length or diameter did, however, did not demonstrate a similar effect on the insertion energy. It has also been noted during the experiments that apical tapering of the implants allowed placing them at different depths into standardized osteotomies prior to engaging the bony walls. Other than for recording maximum insertion torque, which is normally reached during final seating of an implant, this affects the dynamic parameter of insertion energy.

As such, resonance frequency analysis as a measure of primary stability appeared to offer greater benefit in distinguishing the different implant groups. A previous clinical study found that tapered implants required higher insertion torque when compared to straight-walled implants but provided better primary stability. However, the tapered implants also had a lower clinical success outcome, thus implicating the level of insertion torque applied [15]. Despite a study by Elias et al. [6] claiming that the influence of the surgical technique would exert less effect than that of implant size and shape, it had been expected that the application of the dense bone drilling protocol would lead to a reduction in measurement values. When considering maximum insertion torque and insertion energy, this study found the opposite. Consequently, one could conclude that the implant manufacturer’s surgical placement protocol is not suited to avoid ‘overstressing’ dense bone. Notwithstanding, dense bone protocols generally can be expected to provide for less under-sizing of the osteotomy compared to medium and low bone quality drill protocols [6]. As shown in this study, the effect of changing the drill protocol can potentially be monitored using the novel parameter of insertion energy.

In interpreting the data from this study, the authors acknowledge the limited sample size. Two further limitations are noted: Layered polyurethane foam material [2,8,9,19] has been used as a bone surrogate and consequently, the absolute values measured here cannot be directly transferred to clinical reality. Additionally, these materials are not able to fully mimic the elastic properties of alveolar bone, but in turn, allow for reproducible measurements [6]. The process of implant placement strongly depends on the drill protocol used in the preparation of the osteotomy and the design of the implant. As only one specific implant design has been used in this investigation, the results should be interpreted within the scope of the study. Future studies should consider the effect of different implant materials such as zirconia ceramic on the surgical protocols applied [5] as well as the biologic effects associated with a specific level of insertion torque. In addition, measurement parameters directly evaluating mechanical bone quality should be established in contrast to developing implant-related surrogate criteria.

## Figures and Tables

**Table 1:** Description of the four experimental groups investigated.

Group	Implant	Surgical Protocol
Control	ICX 4.8 × 12.5 mm	Medium Bone
Short	ICX 4.8 × 10.0 mm	Medium Bone
Slim	ICX 4.1 × 12.5 mm	Medium Bone
Dense	ICX 4.8 × 12.5 mm	Dense Bone

**Table 2:** Mean values and standard deviations (SD) for all parameters evaluated.

Group	Control		Short		Slim		Dense	
	Mean	SD	Mean	SD	Mean	SD	Mean	SD
Maximum insertion torque [Ncm]	15.9	2.24	15.1	0.85	15.7	1.53	17.2	0.55
Implant Stability [ISQ] *	64.0	1.32	61.0	0.79	62.8	1.40	61.6	1.47
Insertion Energy [Ncm·s] *	819.08	77.78	703.46	46.41	800.36	77.86	973.24	82.56

* significant differences appeared in these parameters.

**Table 3:** Results (*p*-values) of ANOVA and pairwise comparisons between the different implant systems used for all three parameters tested (Tukey’s honest differences test; α = 0.05; significant differences are written in bold).

	Maximum Insertion Torque	Implant Stability	Insertion Energy
ANOVA	0.17	**0.01**	**0.00**
Control vs. Short	0.82	**0.01**	0.09
Control vs. Slim	0.99	0.47	0.98
Control vs. Dense	0.49	**0.04**	**0.02**
Short vs. Slim	0.92	0.16	0.19
Short vs. Dense	0.14	0.88	**<0.01**
Slim vs. Dense	0.36	0.47	**<0.01**

**Table 4:** Pearson product moment correlation for the parameters implant stability quotient (ISQ,) maximum insertion torque and insertion energy (α = 0.05; significant correlations are written in bold).

	ISQ vs. Maximum Insertion Torque		ISQ vs. Insertion Energy		Maximum Insertion Torque vs. Insertion Energy	
	r-Value	*p*-Value	r-Value	*p*-Value	r-Value	*p*-Value
Control	−0.38	0.53	0.09	0.89	0.77	0.13
Short	−0.35	0.56	−0.69	0.20	0.86	0.06
Slim	0.15	0.81	−0.01	0.99	0.68	0.20
Dense	−0.25	0.69	−0.06	0.92	0.78	0.12
Overall	−0.10	0.67	0.02	0.95	0.75	**<0.01**
